# A Charge-Reversal Point Mutation Completely Depletes Flavin Chromophore from European Robin Cryptochrome 4a Protein

**DOI:** 10.1021/acs.jpclett.5c03833

**Published:** 2026-02-16

**Authors:** Jingjing Xu, Emil Sjulstok Rasmussen, Francis Berthias, Jessica Schmidt, Henrik Mouritsen, Ole N. Jensen, Ilia A. Solov’yov

**Affiliations:** † Department of Biochemistry and Molecular Biology, 6174 University of Southern Denmark, Campusvej 55, 5230 Odense M, Denmark; ‡ Department of Biology, 6174 University of Southern Denmark, Campusvej 55, 5230 Odense M, Denmark; ¶ Danish Institute for Advanced Study, 6174 University of Southern Denmark, Campusvej 55, 5230 Odense M, Denmark; ◧ Department of Neuroscience, 12334 University of Texas Southwestern Medical Center, 6000 Harry Hines Blvd., Dallas, Texas 75390-9111, United States; ∥ Institute of Biology and Environmental Sciences, 11233 Carl von Ossietzky Universität Oldenburg, Carl-von-Ossietzky-Straße 9-11, 26129 Oldenburg, Germany; ⊥ Research Centre for Neurosensory Science, 11233 Carl von Ossietzky Universität Oldenburg, Carl-von-Ossietzky-Straße 9-11, 26129 Oldenburg, Germany; # Institute of Physics, 11233 Carl von Ossietzky Universität Oldenburg, Carl-von-Ossietzky-Straße 9-11, 26129 Oldenburg, Germany; ○ Center for Nanoscale Dynamics (CENAD), 11233 Carl von Ossietzky Universität Oldenburg, Carl-von-Ossietzky-Straße 9-11, 26129 Oldenburg, Germany

## Abstract

Cryptochrome 4a (Cry4a) is a magnetically sensitive protein that could enable night-migratory birds to sense the geomagnetic field for navigation. The key to the protein’s magnetic sensitivity is the flavin adenine dinucleotide (FAD) cofactor, which initiates the electron transfer within the protein, leading to a spin-correlated radical pair. Despite its importance, the mechanism of FAD binding in avian Cry4a proteins remains unclear. Here, we show that a point mutation of the positively charged arginine residue at position 356 to a negatively charged glutamic acid (R356E) completely depletes FAD binding from European robin (*Erithacus rubecula*) Cry4a. The result indicates that electrostatic interactions constitute the primary driving force that enables the FAD binding in European robin Cry4a. The finding provides new structural insight into the molecular basis of FAD binding in Cry4 and advances our understanding of the biophysical underpinnings of bird magnetoreception.

## Graphical abstract

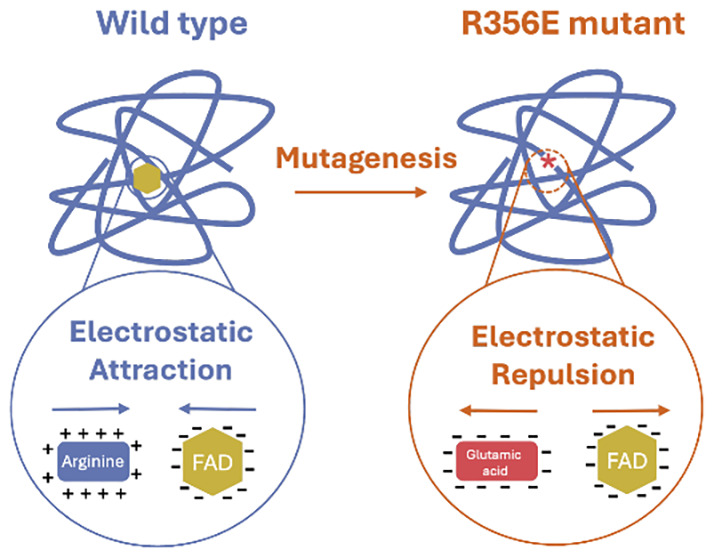

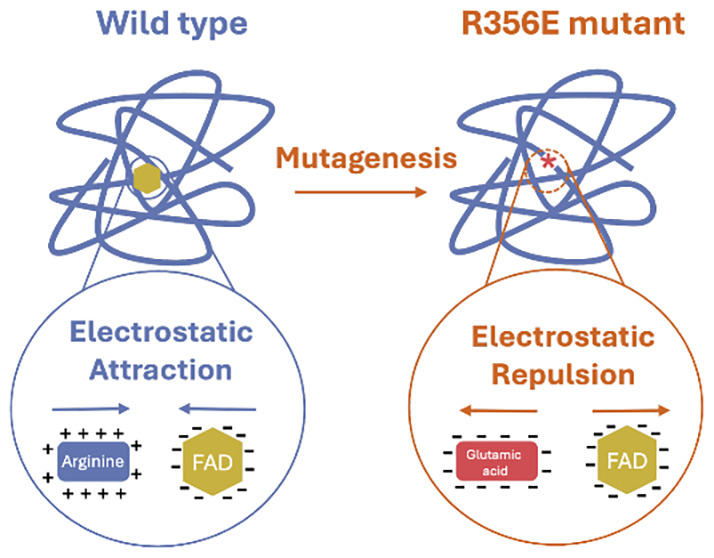

Cryptochrome (Cry) proteins are flavoproteins that are involved in diverse biological processes including photoreception, circadian regulation, and potentially magnetoreception.^1–7^ The diverse biological functions are often associated with the flavin adenine dinucleotide (FAD) cofactor that can be noncovalently bound by some Cry proteins. Essentially, FAD is a blue light absorbing chromophore that can trigger the electron transfer process, leading to a likely biological signaling cascade related to the protein.^8–13^ Animal cryptochromes are classified into Type I, II, and IV. Type I Cry proteins, such as *Drosophila melanogaster* Cry (*Dm*Cry), are blue-light photoreceptors through its FAD binding capacity.^14,15^ Type II Crys function as circadian clock regulators in a light-independent manner and do not bind FAD.^16–19^ Rather, they utilize the primary and secondary binding pockets as protein–protein interaction sites for the circadian proteins CLOCK and PER.^20–22^ Some studies suggest that FAD binding can be induced by saturating Type II Crys with excess FAD.^23^ Type IV Crys, exemplified by avian Cry4a, bind FAD^18,24–26^ noncovalently and tightly. Substantial theoretical and experimental evidence supports that avian Cry4a undergoes photoinduced electron transfer between FAD and a conserved tetrad of tryptophans, a hallmark of radical-pair-based magnetic sensing *in vitro*.^8,18,25,27–30^

Despite the importance of FAD for the light-dependent functioning of the protein, the molecular mechanism of FAD binding in Cry proteins is only partially defined. In *Dm*Cry, single-point mutations hardly deplete FAD binding, and double point mutations only partially deplete FAD binding by around 58%.^19^ Structural analysis suggests that FAD binding in *Dm*Cry is mediated through a cooperative effect of electrostatic interactions and steric modulation of the binding pocket accessibility.^31^ However, so far, there has been no killer experiment to completely deplete FAD from a Cry protein.

Importantly, the lack of a strategy to fully deplete FAD from the Cry4a protein further limits the ability to unambiguously disentangle FAD-dependent signaling processes from protein–protein interaction effects. A Cry4a mutant that is selectively deficient in FAD binding would provide a powerful negative control for functional and mechanistic studies. Such a mutant would enable direct assessment of how the FAD occupancy influences photoreactivity, conformational dynamics, and downstream signaling pathways. Therefore, uncovering the molecular basis of FAD binding in avian Cry4 is critical for rational manipulation of Cry4 function through targeted disruption of cofactor stabilization and an understanding of the light-dependent magnetic sensitivity of Cry4a.

In this study, we use molecular dynamics (MD) simulations to identify key amino acids involved in FAD binding in European robin (*Erithacus rubecula*) Cry4a (*Er*Cry4a). Furthermore, we generate a recombinant *Er*Cry4a mutant protein based on the computational results and experimentally assess FAD binding ability using light absorption spectroscopy and mass spectrometry (MS). Finally, we determine the conformational difference between *Er*Cry4a wild type (WT) and mutant using native electrospray ionization mass spectrometry (MS) and ion mobility spectrometry MS (IMS) and propose a model of the FAD binding mechanism in *Er*Cry4a.

We first analyzed interactions between *Er*Cry4a protein and FAD using Protein–Ligand Interaction Profiler (PLIP), a web tool for identification of noncovalent interactions between biological macromolecules and their ligands.^32^ The result suggests that 11 amino acid residues in *Er*Cry4a could interact with FAD (Figure 1A). The 11 amino acid residues are S245, T246, T247, Q287, W350, H353, R356, F379, D385, D387, and N334. Strikingly, R356 is located parallel to both isoalloxazine and ribitol moieties of FAD and interacts with FAD via diverse interactions, including electrostatic-, cation-π, and hydrogen-bonding interactions. In contrast, S245, T246, and T247 appear to only form a weak hydrogen-bonding network with FAD phosphate groups.

**Figure 1:**
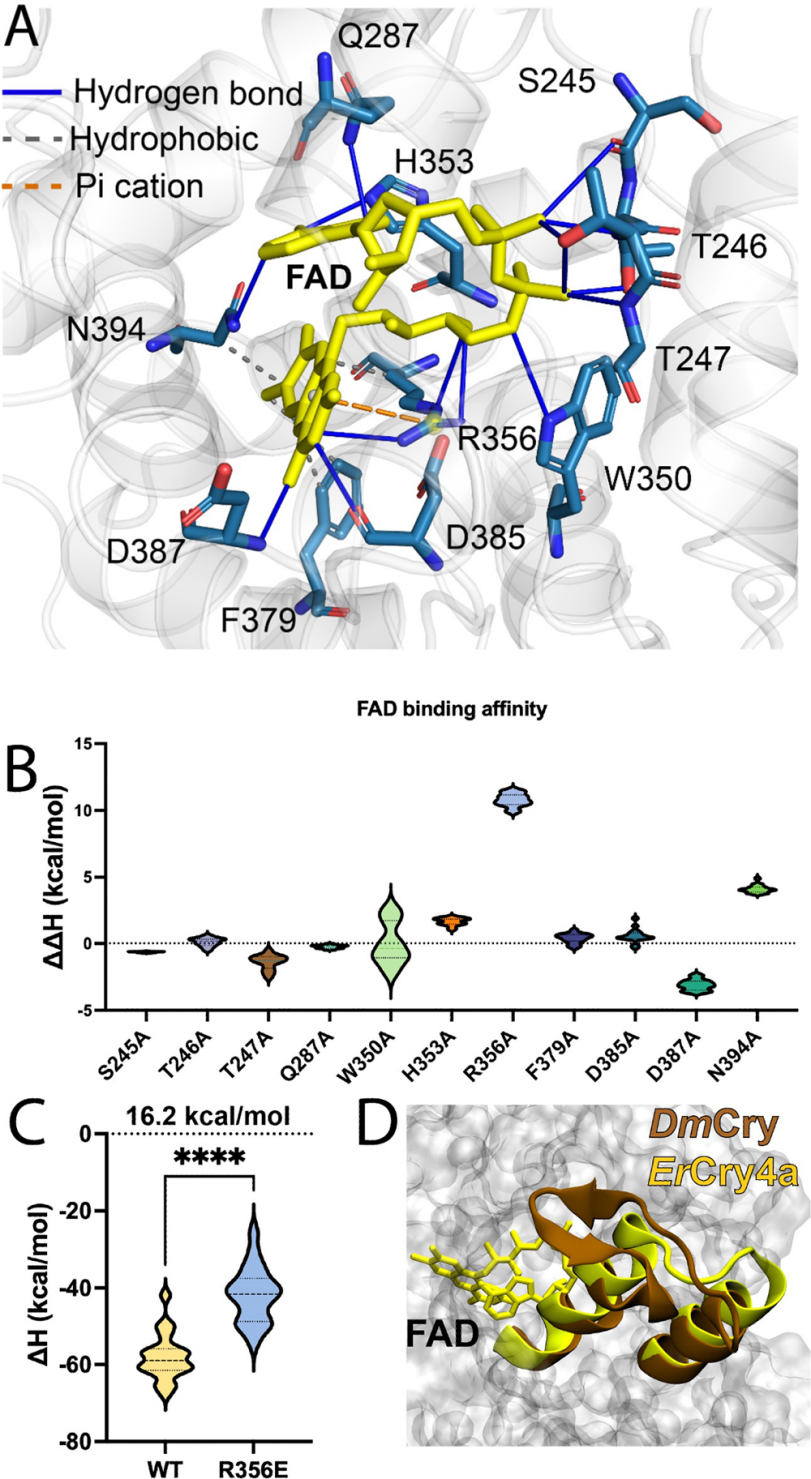
Computational simulation suggests key amino acids that affect FAD binding. (A) Illustration of interactions between amino acid residues and the flavin adenine dinucleotide (FAD) cofactor inside European robin (*Erithacus rubecula*) Cry4a protein (*Er*Cry4a). A total of 11 amino acid residues in *Er*Cry4a potentially interacts with FAD through hydrogen bond, hydrophobic interaction, and cation−π interactions. (B) Predicted changes in the binding free energy upon mutation to alanine for the 11 amino acid residues that interact with the FAD. For the higher ΔΔH values, the mutations are predicted to more significantly disrupt FAD binding. (C) Predicted binding affinity for FAD in *Er*Cry4 wild type (WT) and R356E mutant. The absolute value of ΔH in R356E is 16.2 kcal/mol smaller than that of the WT protein, indicating a weaker FAD binding affinity in R356E compared to that in WT. It should be stressed that the absolute ΔH values cannot be used to directly determine the FAD binding affinity, but these calculations reliably predict changes in binding affinities. (D) Structural comparison of *Er*Cry4a and *Drosophila melanogaster*cryptochrome (*Dm*Cry). The β-sheet on top of FAD in *Dm*Cry is absent in *Er*Cry4a, indicating a negligible steric constraints effect on FAD binding in *Er*Cry4a.

To quantify the interactions between the 11 residues and FAD, we computed the binding free energy of X-to-alanine mutants, where X refers to one of the 11 residues. Alanine scanning mutagenesis is ideal for testing how much a particular residue contributes to binding because alanine is small and chemically neutral.^33,34^ The computational alanine scanning result shows that the R356 residue stands out of the 11 amino acid residues surrounding FAD due to the high binding free energy (10 kcal/mol), indicating that R356 is the most energetically significant contributor to FAD binding (Figure 1B).

We hypothesize that the strong interaction between R356 and FAD is primarily driven by electrostatic forces, as arginine has a positive charge, while FAD is negatively charged. To test the hypothesis, we introduced a charge-reversal mutation, replacing R356 with negatively charged glutamic acid (R356E). Computational simulations suggest that the FAD binding affinity of R356E is 16.2 kcal/mol lower than that of the wild type, indicating substantially weaker FAD binding in the mutant (Figure 1C). Notably, no obvious changes in the electrostatic potential surface map are observed (Figure S1), probably because R356 is buried within the binding pocket. Furthermore, structural analysis reveals that a β-sheet critical for FAD binding in *Dm*Cry is absent in *Er*Cry4a (Figure 1D), suggesting that steric constraints on FAD binding in *Er*Cry4a are negligible.

Following the *in silico* results, we generated the *Er*Cry4a R356E mutant in the wet laboratory through site-directed mutagenesis and recombinant protein expression. During the gradient elution process of anion exchange chromatography, the R356E mutant eluted from the anion exchange column at a solvent conductivity similar to that of the WT protein (Figure 2A), suggesting that R356E mutagenesis maintains a similar surface charge pattern as in the WT protein. The purity of both protein samples is also similar, as shown by the single band on the Coomassie blue staining image of a protein gel (Figure 2B). Most importantly, the UV–vis spectrum of *Er*Cry4a R356E mutant appears flat (near-to-null) in the absorbing range between 300 and 500 nm, indicating no blue-light absorption of R356E mutant and thus no FAD binding (Figure 2C). As a positive control, the WT protein shows vibrational fine structure around 350 and 450 nm, which are characteristic features typically associated with FAD binding in cryptochrome proteins.

**Figure 2:**
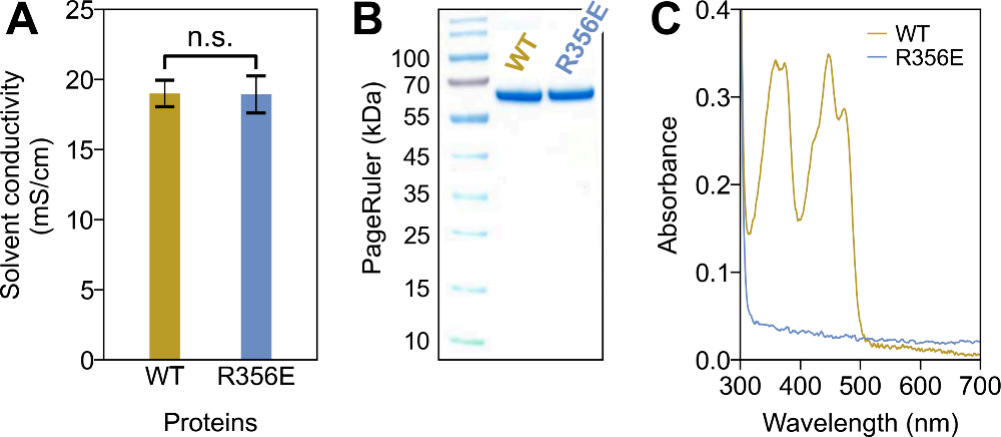
A single-point mutant *Er*Cry4a R356E completely deplete FAD from *Er*Cry4. (A) Both R356E and WT proteins elute from the anion exchange column at similar solvent conductivity during gradient elution process of anion exchange chromatography. (B) Sodium dodecyl sulfate–polyacrylamide gel electrophoresis Coomassie Blue Dye staining suggests that both proteins are comparably pure. (C) UV–visible absorption spectrum shows that the *Er*Cry4 R356E mutant does not absorb blue light between 350 and 500 nm, indicating the absence of FAD (the blue-light chromophore) binding. *Er*Cry4 wild type (WT) is used as a positive control showing characteristic spectral features of FAD binding.

Furthermore, mass spectrometry was employed to determine the protein sequence and cofactor binding status of the *Er*Cry4a WT and R356E mutant. In both denaturing and native MS measurements, the spectra exhibit peak broadening with shoulder features (Figure 3). This broadening is most likely caused by unresolved isotopic distributions and residual adduct heterogeneity (e.g., sodium or acetate adducts) (Figure S2). Importantly, these effects do not compromise the robustness of the measurements, as independent acquisitions recorded under identical conditions show highly consistent spectra, demonstrating good repeatability of the MS analysis (Figure S3).

**Figure 3:**
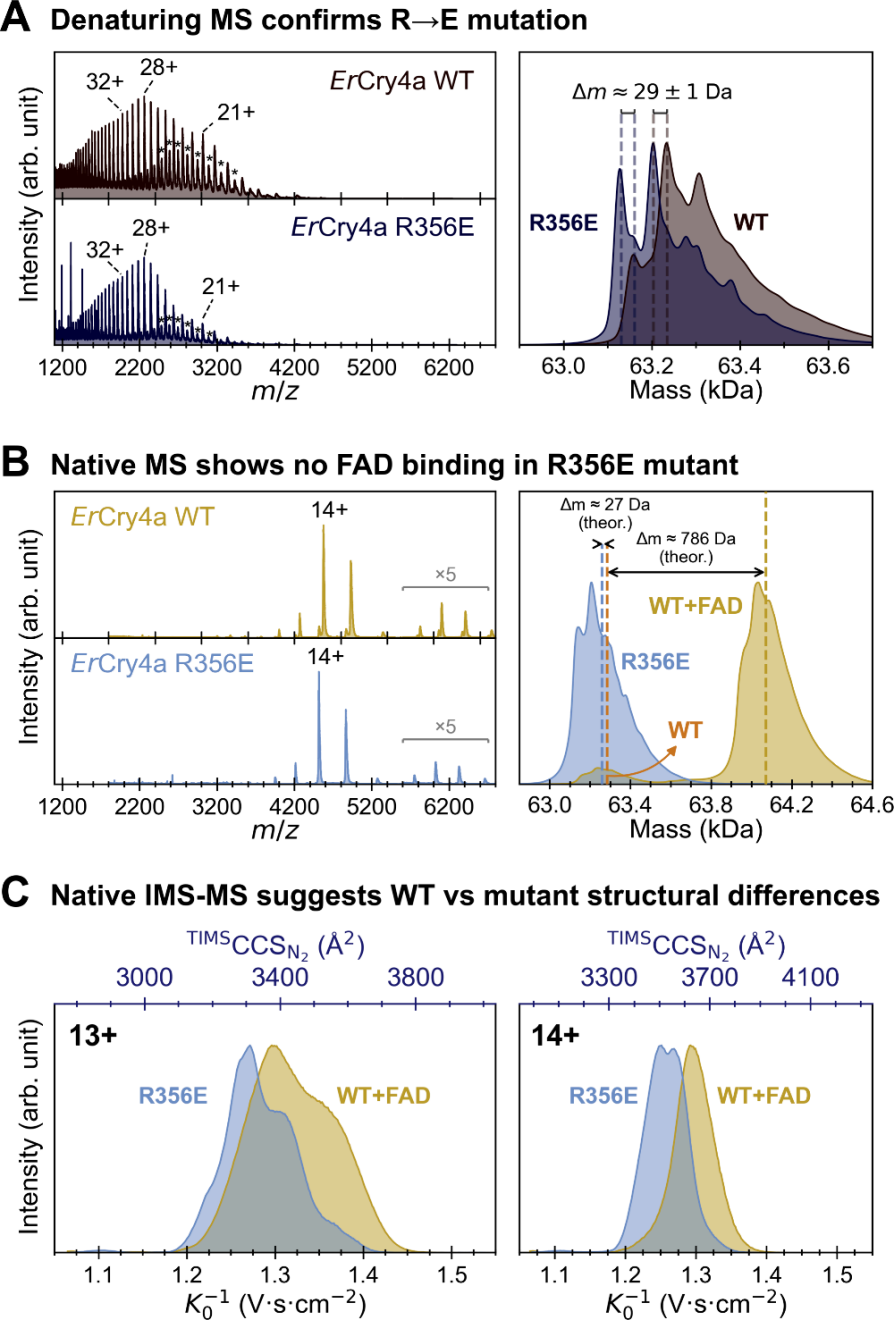
Mass spectrometry confirms amino acid substitution, loss of FAD binding and structural differences in *Er*Cry4a R356E mutant. (A) Denaturing mass spectrometry shows an ∼29 Da mass difference between WT and R356E mutant, consistent with theoretical mass difference between arginine and glutamic acid residues (27.08 Da). The ∼2 Da uncertainty arises from inherent limitations of the UniDec deconvolution algorithm.^36,37^ Protein dimers are marked by stars in the left panel (*m*/*z* range: 2400–3400). (B) Native MS reveals a mass difference of 786 Da between WT and R356E, matching the molecular weight of FAD. Vertical dashed lines are visual guides to indicate the average theoretical expected masses of the apo and FAD-bound species. The deconvoluted mass envelopes are broadened and slightly asymmetric due to residual adduct heterogeneity and minor overlapping contributions, which can lead to deviations between the peak apex and its centroid. The dimer population is highlighted by a 5-fold magnification (*m*/*z* range: 5600–6800). (C) Ion mobility spectrometry–mass spectrometry (IMS-MS) shows a slight left shift in collision cross section for the R356E FAD-free mutant relative to the FAD-bound WT, indicating a more compact conformation of the FAD-free mutant.

Under denaturing conditions, both proteins display broad charge state distributions, ranging from ∼18+ to 46+. The broad charge state distributions suggest that the proteins are completely unfolded, and thus, FAD was removed from the protein. This allowed assessment of the subtle mass difference attributable solely to amino acid substitution. Deconvolution of the denatured spectra yields a mass difference of ∼29 Da between WT and R356E, which matches the theoretical mass difference between arginine and glutamic acid being 27.08 Da (Figure 3A). Consistently, a minor mass shift was also observed in native MS measurements on the FAD-free WT protein and the R356E mutant protein (Figure 3B, the right panel). Together, the MS results validate that arginine has been successfully replaced with glutamic acid in the R356E mutant.

Under native-like conditions, the charge state distributions of *Er*Cry4a WT and mutant protein narrow between 12+ and 16+, dominated by 13+ and 14+, indicating that the proteins are folded as expected. The R356E mutant is detected exclusively without FAD, whereas the majority of the WT (94%) binds FAD, indicated by a 786 Da mass difference (Figure 3B). It is interesting to note that a dimer species is observed in both denaturing (Figure 3A) and native (Figure 3B) measurements. The protein dimers are very likely formed through the covalently disulfide bonds due to the absence of reducing reagent in the buffer solvent. The observation is consistent with our previous study on *Er*Cry4a dimer.^35^

Ion mobility spectrometry (IMS) measurements provide further insight into the conformational features of the two proteins. The R356E mutant protein exhibits a slightly smaller (left-shifted) collision cross section (CCS) value compared to the FAD-bound WT protein (Figure 3C). For example, at the 14+ charge state, the CCS values are 3625 Å^2^ and 3530 Å^2^ for the R356E mutant protein and the FAD-bound WT protein, respectively. Owing to the high intraday measurement repeatability (Figure S3), this CCS difference is considered robust and reproducible. The measured CCS difference (ΔCCS) between the two proteins is 2.7%. By contrast, the CCS change expected solely from replacing arginine with glutamic acid is approximately ∼0.9%, which was calculated as CCS ∝ *m* ^2/3^.^38^ Consequently, the observed substantial CCS shift cannot be explained by residue mass alone but instead reflects a conformational effect associated with cofactor binding. Grounded in the native IMS-MS results and with mass effects excluded as the dominant contributor, we conclude that the R356E mutant protein exhibits a conformation more compact than that of the FAD-bound WT protein. Such large-scale, long-time-scale conformational rearrangements will be an interesting target for future investigation using advanced molecular dynamics simulations.

In summary, our study clearly shows that site-specific electrostatic repulsion is sufficient to completely block the binding of FAD in *Er*Cry4a. To our knowledge, this is the first report of a single-point mutation that completely depletes FAD binding in any Cry protein. Previous work on *Dm*Cry required dual mutations (R298E + Q311E) and the mutations only partially reduced FAD occupancy by 58%.^19^ FAD binding in Cry proteins has been proposed to depend on two factors: (i) noncovalent interactions between FAD and the binding pocket and (ii) the steric availability of the binding pocket itself.^31^ In general, the structure of the FAD binding pocket is rigid. However, in certain situations, major movements can happen, such as seen in *Dm*Cry.^31^ In Type I Cry, strong noncovalent interactions and a conformationally closed binding pocket make FAD depletion difficult, typically requiring multiple mutations—one to weaken the noncovalent interactions and another to open the pocket.^19,31^ Specifically, in the *Dm*Cry double mutant, R298E was used to remove the β-sheet lid and Q311E was used to remove the pincer holding FAD in place.^31^ In the present study, we discovered that FAD binding is primarily mediated by electrostatic attraction, while steric constraints play a negligible role in *Er*Cry4a (Figure 4). It is perhaps surprising that one could achieve complete FAD depletion via a single-point mutation. However, an evolutionary divergence in the FAD-binding mechanism is likely to have occurred between Type IV Cry, exemplified by ErCry4a, and Type I Cry, exemplified by *Dm*Cry, due to differences in their protein structures. The apparent absence of a canonical β-sheet lid in *Er*Cry4a raises broader questions about how Cry proteins have been structurally diversified to support species-specific physiological functions.

**Figure 4:**
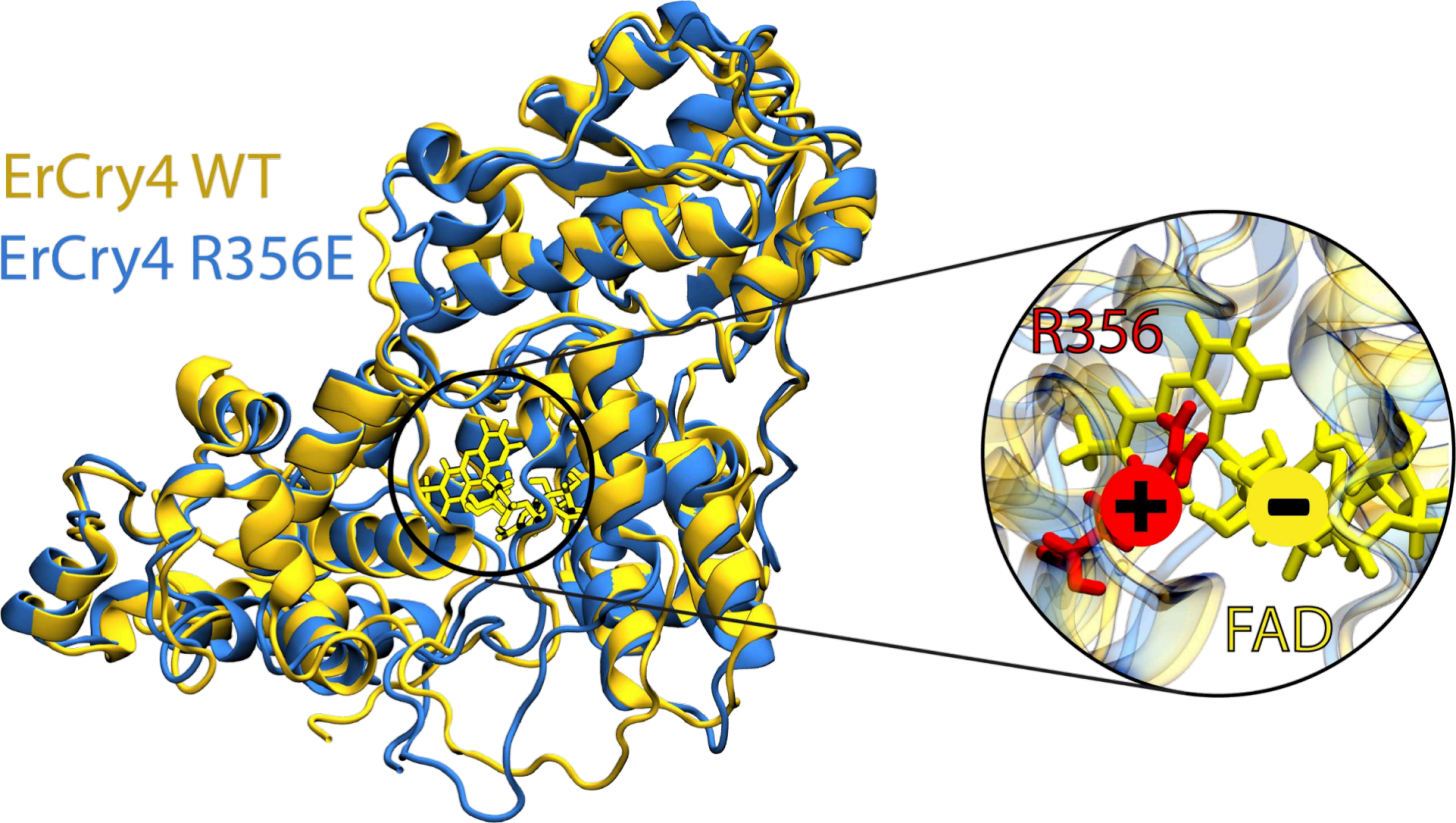
A model of FAD binding mechanism in *Er*Cry4a protein: electrostatic attractions secure FAD noncovalently binding in Cryptochrome 4a. The modeled structure of *Er*Cry4a WT and R356E mutant are shown in yellow and blue, respectively. The inset shows the interaction interface between FAD and the R356E residue.

Single-point mutations are generally preferred in functional studies, as they introduce minimal perturbations to the protein structure compared to dual or multiple mutations. In the present study, the single-point mutation preserved the overall charge profile of *Er*Cry4a WT protein, with only a slightly more compacted conformation observed in the mutant. The FAD-free R356E mutant of *Er*Cry4a will serve as an ideal negative control to investigate the structural and functional role of FAD in cryptochrome signaling and protein–protein interactions.^39^ In a much broader context, flavin-bound proteins have recently been employed as photosensitizers in photocatalytic proximity labeling, a technique that uses light-activated catalysts to tag nearby proteins or molecules for identification.^40^ A flavin-free Cry4a mutant can also provide a valuable negative control in this emerging application. In this regard, our study establishes a framework for the generation of flavin-free cryptochrome apoproteins. Such a charge-reversal strategy is probably applicable to Cry4a proteins from multiple bird species as the arginine residue is highly conserved across avian Cry4a sequences (Figure S4), suggesting a general and transferable mutagenesis approach.

In conclusion, this study provides new molecular insight into cofactor stabilization in avian Cry4a, revealing that electrostatic interactions rather than steric constraints dominate FAD retention. The R356E mutant serves as a powerful negative control for functional studies of *Er*Cry4a, enabling precise dissection of FAD-dependent signaling pathways in magnetoreception and in emerging photocatalytic proximity labeling applications. Future work should investigate whether the charge-reversal single-point mutagenesis strategy similarly disrupts FAD binding in Cry4a proteins from other bird species and how FAD binding influences Cry4a conformational dynamics and protein–protein interactions,^9–11^ which are both critical for understanding its role in light-dependent magnetic sensing.^7^

## Methods

A homology model of *Er*Cry4a was built based on the *Cl*Cry4 template (PDB ID: 6PU0)^18^ in Swiss model workspace. The resulting *Er*Cry4a homology model was solvated in a water box with a padding distance of 12 Å. Hydrogens were added to the *Er*Cry4a model, while the topology was created using pdb 2gmx from the gromacs software.^41,42^ The protonation state of *Er*Cry4a was estimated at pH 7 using PDB 2PQR^43^ from the APBS biomolecular solvation software suite Web site.^44^ FAD was parametrized using GAFF2,^45^ while the *Er*Cry4a protein was simulated using the ff99SB-ILDN force field.^3^ Specifically, simulations were carried out using a 2 fs time step with the leapfrog integrator. Energy minimization prior to the molecular dynamics simulation was carried out using the steepest descent method. The temperature of the system was set to 300 K using the modified Berendsen thermostat, and pressure was controlled at 1 bar using the Parrinello–Rahman barostat.^46,47^ Long range electrostatic interactions were modeled using the Particle mesh Ewald summation approach.^48^ Constraints were set on hydrogen bond distances using the LINCS algorithm.^49^ Starting velocities were generated with a random seed. The simulation protocol included (i) energy minimization for 20,000 steps, (ii) equilibration with restraints on the protein in the constant-temperature ensemble for 10 ps, (iii) equilibration in the constant-pressure ensemble for 100 ps, and (iii) production simulation in the constant-pressure ensemble for 50 ns. The residues that potentially influence FAD binding affinity were scanned using the protein ligand interaction profiler Web server.^32^ Here, the structure used to profile the protein ligand interaction is a relaxed shot following MD simulations. The redox state used for flavin is the oxidized state. The *in silico* alanine scanning technique was subsequently used to quantify the contribution of the residues to the FAD binding affinity. Constant-pressure simulations were carried out following the equilibration, and 20 frames were prepared as the input for the alanine scan analysis. Mutations of the FAD-surrounding amino acid residues to alanine were carried out using gmx_MMPBSA.^50^ FAD binding affinities for *Er*Cry4a wild type and mutant proteins were calculated using the Poisson–Boltzmann method implemented in gmx_MMPBSA.^50^ The analysis was carried out in Prism using Welch’s *t*-test to find significance and differences in binding affinities.

*Er*Cry4a R356E mutant DNA was generated through a polymerase chain reaction (PCR) using the Q5 site-directed mutagenesis kit (New England Biolabs, Ipswich, MA, USA). The PCR primer sequences employed were 5′-TCA­CCT­GGC­TGA­ACA­CGC­CGT­CG-3′ and 5′-TGG­ATC­CAG­CCT­TCC­TGG-3′. The DNA sequence was confirmed via Sanger sequencing (LGC Genomics, Berlin, Germany). The *Er*Cry4a R356E mutant and the WT protein were expressed and purified as previously reported.^25,27^ The UV–visible absorption spectra were measured using a Cary 60 UV–vis spectrophotometer (Agilent, Santa Clara, CA, US).

Twenty μM protein was used for MS measurements. Prior to the native MS measurements, protein samples were buffer-exchanged to 150 mM ammonium acetate (pH 8) using ZebaTM Micro Spin desalting columns with a molecular weight exclusion limit of 40 kDa (ThermoFisher Scientific). Prior to the denaturing MS measurements, proteins were incubated in pH 2 150 mM ammonium acetate solution for 5 min. The MS and ion mobility spectrometry–MS (IMS-MS) data were acquired on a timsTOF Ultra1 instrument (Bruker Daltonics) in positive ion mode using a CaptiveSpray 2 nanoESI source. To generate an ion source, the capillary voltage was set to 1.1 kV and dry gas was pumped at a speed of 3 L min^–1^. The general temperature of the ion source was 80 °C. For the ion optics (native transmission), the parameters were as follows: Deflection 0 Delta = 105 V, Funnel 0 RF = 250 Vpp, Multipole 0 RF = 200 V, Deflection 1 Delta = 80 V, Funnel 1 RF = 400 Vpp, isCID Energy = 100–125 eV, Funnel 2 RF = 600 Vpp, Multipole RF = 1200 Vpp, Collision energy: 10 eV, Collision RF: 2000 Vpp, Ion Energy = 10 eV, Low Mass = 500 m/*z*, Transfer time = 120 μs, Prepulse storage = 120 μs.

The trapped ion mobility spectrometry in/out tunnel pressures used were 2.4 and 0.7 mbar (ΔP 1.7 mbar). The exact parameters were: Accumulation = 20 – 30 ms; 1/*K*_0_ ramp= 100 ms; 1/*K*_0_ window = 0.6 V·s·cm^–1^; scan ramp rate = 1.1 kV·s^–1^. DC potentials: DC potentials: Δ*t*_1_ = −20 V, Δ*t*_2_ = −120 V, Δ*t*_4_ = 100 V, Δ*t*_5_ = 0 V, and Δ*t*_6_ = 20–40 V·m/*z* and 1/*K*_0_ were calibrated with an ESI-L low-concentration tune mix (Agilent). The collision cross section (CCS) in N^2^ were calculated from the Mason–Schamp equation using the manufacturer CCS values. The MS and IMS-MS spectra were exported using Compass DataAnalysis software (Bruker Daltonics, v6.1). The deconvoluted spectra were obtained using UniDec^37^ with the following parameters: *m*/*z* ratio range: 1500–5000 Th, charge range: 10–50, mass range: 30–150 kDa, sample mass set to every 5 Da, peak fwhm: 2 Th, peak shape: Gaussian.

## Supplementary Material



## Notes

Electrostatic potential surface maps of *Er*Cry4a WT and R356E mutant proteins, zoomed-in view of the WT protein spectrum under denaturing conditions, intraday repeatability of inverse mobility measurements for *Er*Cry4a WT and R356E, and sequence alignment of cryptochrome 4a proteins from multiple bird species (PDF)
