# Supplementary material for: A Charge-Reversal Point Mutation Completely Depletes Flavin Chromophore from European Robin Cryptochrome 4a Protein
Source: J Phys Chem Lett. 2026 Feb 16;17(13):3742–8. doi: 10.1021/acs.jpclett.5c03833 (PMC13051451; doi:10.1021/acs.jpclett.5c03833)
Supplement: Supplementary file 1 [file jz5c03833_si_001.pdf]

Supplementary Information for

A Charge-reversal Point Mutation Completely Depletes Flavin Chromophore from European Robin Cryptochrome 4a Protein

Jingjing Xu,[*,†,‡,¶,△] Emil Sjulstok Rasmussen,[§,△] Francis Berthias,[†] Jessica Schmidt,[‖] Henrik Mouritsen,[‖,⊥] Ole N. Jensen,[†] and Ilia A. Solov'yov[#,⊥,@]

†Department of Biochemistry and Molecular Biology, University of Southern Denmark, Campusvej 55, 5230 Odense M, Denmark

‡Department of Biology, University of Southern Denmark, Campusvej 55, 5230 Odense M, Denmark

¶Danish Institute for Advanced Study, University of Southern Denmark, Campusvej 55, 5230 Odense M, Denmark

§Department of Neuroscience, University of Texas Southwestern Medical Center, 6000 Harry Hines Blvd. Dallas, TX 75390-9111, USA

‖Institute of Biology and Environmental Sciences, Carl von Ossietzky Universit¨at Oldenburg, Carl-von-Ossietzky-Straße 9-11, 26129 Oldenburg, Germany

⊥Research Centre for Neurosensory Science, Carl von Ossietzky Universit¨at Oldenburg, Carl-von-Ossietzky-Straße 9-11, 26129 Oldenburg, Germany

#Institute of Physics, Carl von Ossietzky Universit¨at Oldenburg, Carl-von-Ossietzky-Straße 9-11, 26129 Oldenburg, Germany

@Center for Nanoscale Dynamics (CENAD), Carl von Ossietzky Universit¨at Oldenburg, Carl-von-Ossietzky-Straße 9-11, 26129 Oldenburg, Germany

△These authors contributed equally to this work

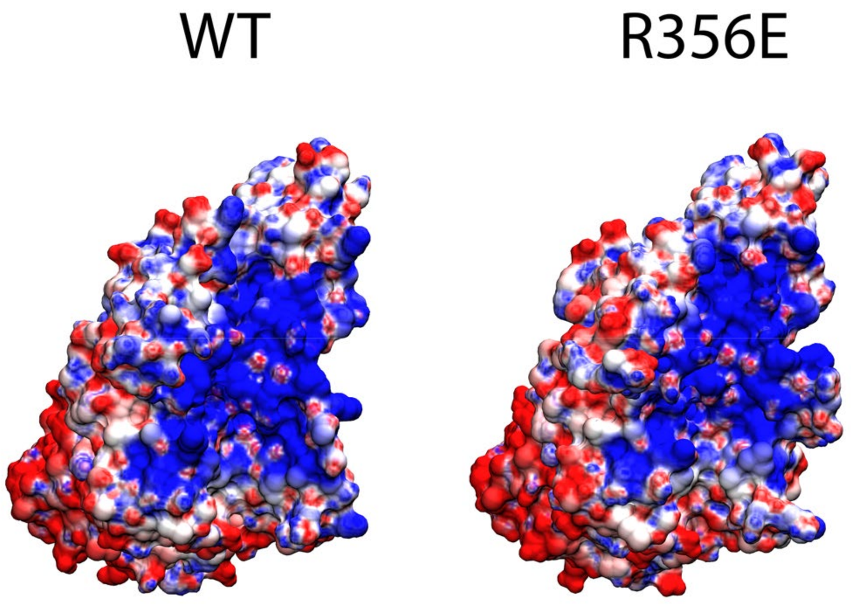

Figure S1 The electrostatic potential surface maps of *Er*Cry4a wild type (WT) and R356E mutant proteins.

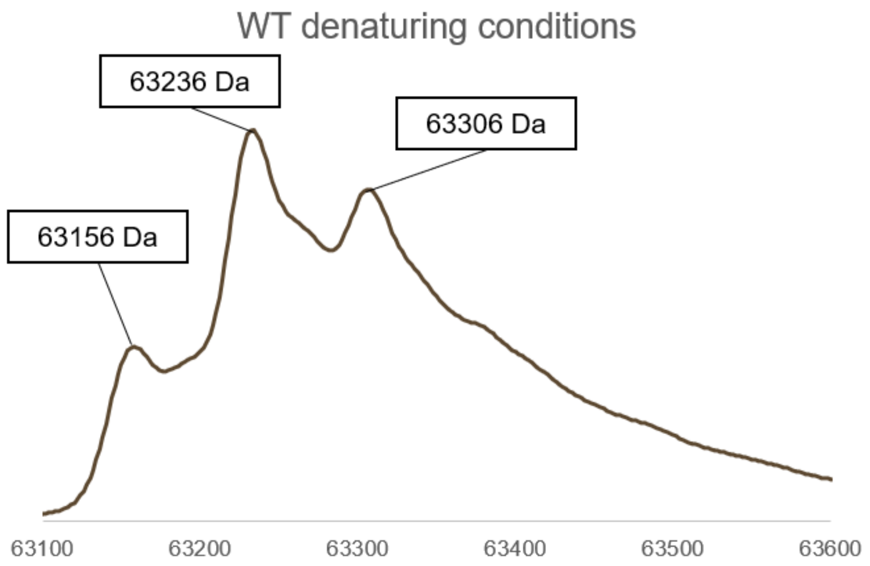

Figure S2 A zoomed-in view of the WT protein spectrum under denaturing conditions. The mass differences are approximately 70–80 Da between the different peaks, which seems too small to correspond to truncation of a single amino acid residue. These differences are instead consistent with residual adducts, for example ~70 Da corresponding to three sodium adducts (3 × 23 Da), and ~80 Da potentially arising from a sodium + acetate combination (≈82 Da).

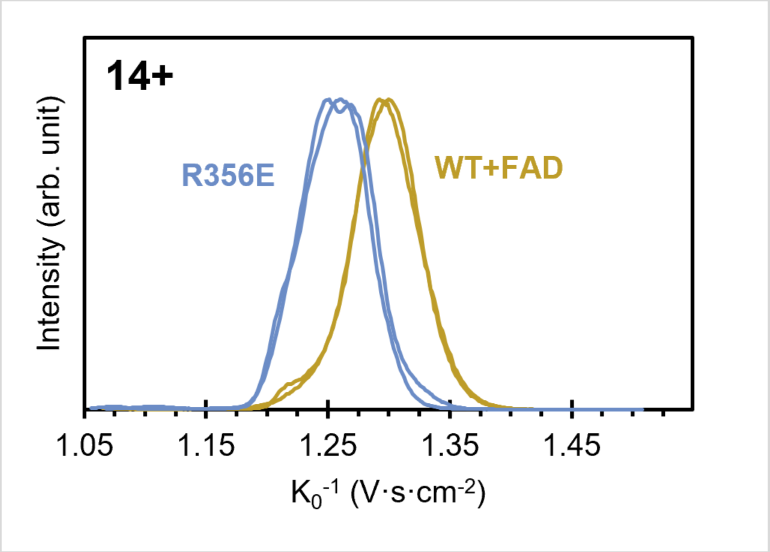

Figure S3 Intra-day repeatability of inverse mobility measurements for *Er*Cry4a WT and R356E. The two independent inverse mobility acquisitions of the 14+ charge state of *Er*Cry4a WT and the R356E mutant were recorded under identical experimental conditions on the same day and show highly consistent distributions, demonstrating good intra-day repeatability of the measurements.

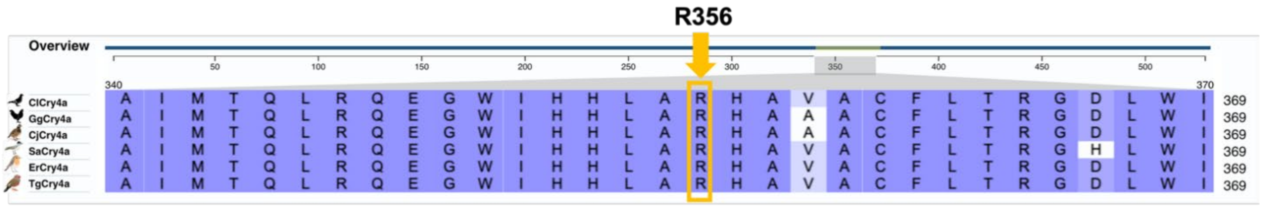

Figure S4 Sequence alignment of cryptochrome 4a proteins from multiple bird species. The arginine residue is conserved across all selected avian cryptochrome 4a sequences, supporting the feasibility of applying the same mutagenesis strategy to generate FAD-free Cry4a proteins in different bird species.
